# The Role and Influence of Gut Microbiota in Pathogenesis and Management of Obesity and Metabolic Syndrome

**DOI:** 10.3389/fendo.2014.00047

**Published:** 2014-04-07

**Authors:** Parth J. Parekh, Eli Arusi, Aaron I. Vinik, David A. Johnson

**Affiliations:** ^1^Department of Internal Medicine, Eastern Virginia Medical School, Norfolk, VA, USA; ^2^Endocrinology Division, Department of Internal Medicine, Eastern Virginia Medical School, Norfolk, VA, USA; ^3^Gastroenterology Division, Department of Internal Medicine, Eastern Virginia Medical School, Norfolk, VA, USA

**Keywords:** microbiota, obesity, metabolic syndrome, insulin resistance

## Abstract

The obesity epidemic has drastically impacted the state of health care in the United States. Aside from poor diet hygiene and genetics, there are many other factors thought to play a role in the emergence of obesity and the metabolic syndrome. There has been a paradigm shift toward further investigating the gut microbiota and its implications in the pathogenesis of a variety of disease states, including inflammatory bowel disease, *Clostridium difficile*, and most recently obesity and the metabolic syndrome. This article is intended to evaluate the role of gut microbiota in the pathogenesis of obesity and metabolic syndrome and its influence in future management.

## Introduction

The obesity epidemic has spread to more than 1/3 of the adult population in the United States. The estimated annual cost of obesity and obesity-related disease in the United States was $147 billion dollars in 2008, approximately $1,429 higher compared to those of normal weight (1). Obesity-related disease such as atherosclerosis, diabetes mellitus, non-alcoholic fatty liver disease, and certain types of cancer are the leading causes of preventable death in the United States. Recent insights have suggested that microbiota play a crucial role in pathogenesis of the metabolic syndrome. As a result, we are in the midst of a paradigm shift in our approach to battling the obesity epidemic. This article illustrates the underlying pathophysiology of obesity and metabolic syndrome as it relates to the gut microbiome, and potential intervention for treatment.

## Microbiota and Obesity: What is the Link?

The gut microbiota is thought to consist of approximately 10^14^ bacteria, which in its entirety is estimated to contain 150-fold more genes than our host genome (2). Recent genetic investigations have revealed that transmissible and modifiable interactions between the microbiome and diet hygiene influence host biology (3).

In 2005, Bäckhead and colleagues sought to investigate the role of the microbiome in germ-free mice (4). The investigators found that alteration of the gut microbiome in germ-free mice with microbiota harvested from conventionally raised, genetically obese mice resulted in a 60% increase in body fat, and the development of insulin resistance within 2 weeks irrespective of reduced consumption (by 29%) and increased activity (by 27%) when compared to germ-free mice whose microbiome was unaltered. Subsequently in 2006, Turnbaugh et al. confirmed these results and in addition found that this trait to be hereditarily transmissible (5). The investigators found that transfer of microbiota from conventionally raised mice with a genetic pre-disposition to into germ-free mice resulted in phenotypically obese mice. Recently in 2013, Riduara et al. sought to establish the relationship between structural and functional configurations of the human microbiome and the resultant disease phenotype (3). In order to do, the investigators transplanted germ-free mice with fecal microbiota from each member of four discordant twin pairs, one from an obese (*ob*) or lean (*ln*) co-twin. The investigators found those communities with microbiota from *ob*-twins were correlated with differences in fermentation of short-chain fatty acids (SCFAs) (increased in the *ln* populous), metabolism of branched chain amino acids (increased in the *ob* populous), and microbial transformation of bile acid species (increased in the *ln* populous) with a net result of an increase in body mass and adiposity in the *ob* subset as compared to its *ln* counterpart.

## Regulation of Host Energy Balance and Storage by Gut Microbiota

As discussed by Riduara et al. (3), a number of underlying mechanisms are thought to coalesce within the host microbiome resulting in obesity. This section will dissect each underlying mechanism as it relates to the microbiota and its development in obesity and the metabolic syndrome.

### Energy extraction from diet

The composition and metabolic actions of the microbiota play a significant role in energy processing of dietary intake. In 2011, Jumpertz et al. evaluated the role of microbiota in regulating nutrient absorption by pyrosequencing bacterial rRNA genes in the feces of 12 lean and 9 obese individuals (6). Additionally, invested calories were measured and compared to stool calories with use of bomb calorimetry. The investigators found that alteration of nutrient load induced changes in the microbiota, which directly correlated with stool energy loss an increased energy harvest of approximately 150 kcal, which led to the conclusion that microbiota may play a substantial role in the regulation of the nutrient harvest.

### Bacterial fermentation and its role in energy harvest

The metabolites of dietary polysaccharides, namely monosaccharides and SCFAs, are produced by the hydrolysis and fermentation carried out by gut microbiota. These metabolites are absorbed and act as an energy source by the host. In 2012, Lin et al. evaluated the effects of SCFA administration in mice with the premise being that SCFAs regulate gut hormones via free fatty acid receptors 2 (FFAR2) and 3 (FFAR3) ultimately protecting against diet-induced obesity and the development of insulin resistance (7). They found SCFAs, namely butyrate and propionate, to induce gut hormones and to reduce overall intake independently via FFAR3. The authors concluded stimulation of gut hormones and curbing of dietary intake via butyrate and propionate to be a novel mechanism by which the microbiota regulates host metabolism.

Microbial fermentation is a complex process resulting in the production of SCFAs. Methanogens in the gut are thought to play a pivotal role in fermentation and ultimately productions of SCFAs with the net result being energy harvest and weight gain (8). In 2006, Samuel and Gordon sought to evaluate the extent to which Archea impact digestive health and were able to demonstrate an impact on host energy harvesting via bacterial utilization of polysaccharides and ultimately the production of SCFAS (9).

Conterno et al. analyzed the fermentation activity of gut microbiota and found it to be increased in obesity (10). Subsequently, Schwiertz and colleagues quantified fecal SCFAs in lean (BMI = 18.5–24.9 kg/m^2^; *n* = 30), overweight (BMI = 25–30 kg/m^2^; *n* = 35), and obese (BMI = >30 kg/m^2^; *n* = 33) volunteers (11). The investigators found the concentration of fecal SCFAs to be significantly greater in the obese cohort (103.9 ± 34.3 mmol/l) as compared to the overweight (98.7 ± 33.9 mmol/l) and lean (84.6 ± 22.9 mmol/l) subjects, which led them to conclude there to be a greater production of SCFA in obese and overweight individuals.

### Suppression of fasting-induced adipocyte factor

Lipoprotein lipase (LPL) plays a pivotal role in hydrolyzing triglycerides and releasing fatty acids for transport into adipocytes. Upon entering adipocytes, these fatty acids are re-esterified into triglycerides and stored as fat. Secreted by adipose, intestine, and liver, angiopoietin-like 4 [fasting-induced adipocyte factor (Fiaf)] antagonizes the activity of LPL, thus preventing storage of triglycerides as fat (8). Bäckhead and colleagues demonstrated an increase in LPL activity in adipose tissue by 122% and simultaneous decrease in Fiaf expression with a net result being an increase in body fat upon conventionalization of germ-free mice (4). Subsequently, Bäckhead et al. evaluated the effect of Fiaf on limit catabolism by comparing the susceptibility of germ-free Fiaf-deficient mice to germ-free wild-type mice (12). The germ-free Fiaf-deficient mice were not resistant to western diet-induced obesity in comparison to germ-free wild-type mice. The investigators were able to demonstrate a model in which the gut microbiota suppresses Fiaf expression in response host sensitivity to over nutrition, thereby increasing LPL activity and ultimately fat deposition in adipocytes.

### Suppression of adenosine monophosphate-activated protein kinase

Adenosine monophosphate-activated protein kinase (AMPK) is an enzyme that plays an active role in energy homeostasis. It is expressed primarily by brain, liver, and skeletal muscle in response to an in AMP:ATP or NAD:NADH ratios, which indicate metabolic stress. As a result, AMPK acts to offset the energy deprived state by stimulating fatty acid oxidation, ketogenesis, glucose uptake, and insulin secretion while inhibiting cholesterol synthesis, lipogenesis, and triglyceride synthesis (13).

Bäckhead and colleagues demonstrated that conventionalization of germ-free mice resulted in a reduced expression of AMPK (12). In particular, AMPK expression in the skeletal tissue of conventionalized mice was markedly reduced when compared to their germ-free counterpart. Therefore, when both populations were fed a western diet, the germ-free population exhibited increased fatty oxidation, which may attribute to their lean phenotype despite exposure to a western diet. In addition, the investigators noted significantly elevated levels of AMP and NAD^+^ in skeletal muscle and liver, respectively, in the germ-free populations. As a result, the investigators concluded that the microbiome has a suppressive effect on AMPK activity with a downstream effect on fatty acid oxidation, thereby predisposing the host to obesity and insulin resistance (12).

### Interaction between SCFAs and G-protein-coupled receptors

As previously discussed, carbohydrate fermentation results in the production of SCFAs, which ultimately results in the regulation of gut hormones such as glucagon-like peptide (GLP) and peptide YY (PYY). These gut hormones are responsible for satiety through regulating the production and release of digestive enzymes (10). Pharmacological and genetic approaches have revealed that the Y-2 receptor mediates the anorectic effects of PYY3–36 (14). Recent studies in rodents have identified the hypothalamus, vagus, and brainstem regions as potential sites of action (15). Using functional brain imaging techniques in humans, PYY3–36 was found to modulate neuronal activity within hypothalamic and brainstem regions involved in reward processing. Thus, the sequence inducing overeating behavior would be alteration of the microbiota and the inhibition of secretion of PYY3–36. Several lines of evidence suggest that low circulating PYY concentrations predispose toward the development and maintenance of obesity (16). Subjects with reduced postprandial release exhibit lower satiety and circulating PYY levels that correlate negatively with markers of adiposity. In addition, mice lacking PYY are hyperphagic and become obese. Conversely, chronic PYY3–36 administration to obese rodents reduces adiposity. Transgenic mice with increased circulating PYY are resistant to diet-induced obesity. The retained responsiveness of obese subjects to the effects of PYY-36 suggests that targeting the PYY system by alteration of the microbiome may offer a therapeutic strategy to help treat obesity.

The signaling cascades of SCFAs are mediated by G protein-coupled receptors, namely FFAR2 and FFAR3. Propionate and butyrate have an affinity for FFAR2, whereas acetate appears to have an affinity for FFAR2 (17). The role of FFAR2 is predominantly to promote energy storage by stimulating adipogenesis, inhibiting lipolysis and decreasing energy expenditure (8). In the colon, both FFAR2 and FFAR3 work in tandem to regulate intestinal motility and satiety via GLP-1 (18). Bjursell and colleagues noted FFAR2-deficient mice (Gpr43^−/−^) who were exposed to a high-fat diet had significantly lower body fat mass, increased lean body mass, greater insulin sensitivity, and lower triglyceride and cholesterol levels than their wild-type counterparts (19). Histologically, the investigators noted a decrease lipid interspersed in brown adipose tissue of the FFAR2-deficient mice while ultimately resulted in higher energy expenditure and higher core body temperature leading to the conclusion that FFAR2 has a protective effect against obesity and dyslipidemia via increased energy expenditure. Samuel et al. evaluated the effect of gut microbiota on adiposity by observing mice deficient in FFAR3 (Gpr41^−/−^) in comparison to wild-type (Grp41^+/+^) (20). The investigators noted that FFAR3-deficient mice had significantly lower body fat mass and increased lean body mass in comparison to their wild-type counterpart, which the investigators attributed to reduced expression of PYY in FFAR3-deficient mice. The downstream effect of this is the stimulation of gut motility and increased energy expenditure coupled with reduced food intake thereby creating a dual effect on energy balance. Increased expenditure and reduced intake generated by an altered microbiome. This led the investigators to conclude that FFAR3 is a regulator of host energy homeostasis through effects that are microbiota dependent. The dual effect is likely to have a greater impact than current medications targeting only one modality.

### Regulation of lipogenesis

Conventionalization of germ-free mice results in a drastic increase in hepatic lipogenesis, a process by which excess glucose is converted to lipids for storage (8). In conventionalized germ-free mice, increased glucose intake and subsequent absorption leads to the activation of carbohydrate response element-binding protein (ChREBP), acetyl-CoA carboxylase (Acc1), fatty acid synthase (FAS), and sterol response element-binding protein-1 (SREBP-1), which in turn increases lipogenesis and insulin concentration (8, 10). It is important to further distinguish the pathways that result in lipogenesis versus that resulting in a negative caloric balance as potential for therapy. Go et al. recently postulated that gut microbiota producing t10,c12 conjugated linoleic acid induced lipogenesis (21). There was a marked increase in lipid accumulation via enhanced incorporation of acetate, palmitate, oleate, and 2-deoxyglucose into triglycerides as well as an increased mRNA expression and protein levels of lipogenic genes, which include SREBP-1, ACC1, FASN, ELOVL6, GPAT1, and DGAT1. This led the authors to conclude that gut microbiota production of t10,c12, conjugated linoleic acid activates *de novo* lipogenesis and triglyceride synthesis resulting in lipid accumulation and increased hepatic steatosis.

### Effect of impaired innate immunity

Toll-like receptors (TLRs) are a type or pattern recognition receptor, which work in concert with Interleukin-1 receptors to form a receptor superfamily known as the “interleukin-1 receptor/TLR superfamily.” Recent studies have targeted TLRs detrimental role in diabetes and its complications. TLRs have since been implicated in the pathogenic process of diabetes via increased blood sugar and non-esterified free fatty acids, and release of cytokines and reactive oxygen species resulting in a pro-inflammatory state that manifests diabetes (22). Toll-like receptor 5 (TLR5) is a protein, which plays a pivotal role in the activation of innate immunity through pathogen recognition via microbe-associated molecular patterns (MAMPs) expressed on bacteria, viruses, and fungi (23). The recognition of PAMPS by TLR5 results in the induction of inflammatory cascades and downstream transcription of various inflammatory cytokines and mediators. Thus, the interaction between microbiota and TLR5 is vital in intestinal homeostasis (8).

The majority of intestinal epithelial cell lines are responsive to flagellin, for which TLR5 has a high affinity. In response to flagellin, TLR5 includes the inflammation cascade via a number of transcription factors, most notably NFκB, in order to enhance host defense and improve survival (24). Vijay-Kumar et al. demonstrated that TLR5-deficient mice (TLR5^−/−^) were prone to developing hallmark features of metabolic syndrome including insulin resistance, hypertension, and hyperlipidemia (25). In addition, TLR5-deficient mice exhibited hyperphagia and as a result developed increased adiposity. Transfer of microbiota from TLR5-deficient mice to its wild-type germ-free counterpart resulted phenotypic manifestation of metabolic syndrome. Interestingly of note, the investigators demonstrated that food restriction in the TLR5-deficient subset prevented obesity, however insulin resistance remained unaffected. The investigators concluded that the gut microbiota contributes to the metabolic syndrome and that malfunction of the innate system may further promote its development.

## Altering Host Microbiome: A Potential Cure?

Understanding the effect of the intestinal microbiome on the pathogenesis of metabolic syndrome and insulin is of utmost importance in developing alternative approaches to therapy. It is also important to isolate specific microbes that may contribute to obesity, and therefore be a potential for targeted therapy. Let et al. analyzed rRNA sequences from genetically obese mice, lean mice, wild-type siblings, and their mothers, all of whom were fed the same diet (26). The investigators found that the mouse and human microbiota compositions to be quite similar, with *Firmicutes* and *Bacteroides* predominating the gut flora. The investigators also noted that genetically predisposed mice had a 50% reduction in *Bacteroites* with a proportional increase in *Firmicutes*, which led to the conclusion that the diversity of the gut microbiota is affected by obesity and manipulation of the flora may be useful in regulating energy homeostasis in the obese population. This section will delve into potential therapies assessing the specific impact on specific microbiota, global microbiome as well as energy homeostasis.

### Diet

Diet has recently been implicated in its influence on the gut flora, which led investigators to further evaluate whether diet is solely responsible for the gut microbiota, irrespective of obese phenotype. Hildebrandt et al. investigated the effect of the host phenotype, genotype, immune function, and diet on the gut microbiome (27). The investigators switched lean wild-type and RELMbeta knockout mice from a standard diet to a high-fat diet and noted that wild-type mice became obese whereas the RELMbeta knockout mice remained comparatively lean. The investigators found a significant decrease in *Bacteroides* and an increase in *Firmuctes* in both subsets, which implicating the diet as a source of change in the gut flora and not the obese phenotype, which led to the conclusion that diet is of importance as a determinant of gut microbiome composition. Subsequently, Fleissner et al. evaluated the influence on various diets on the gut microbiota (28). The investigators exposed germ-free and conventional mice to either a low-fat diet (carbohydrate–protein–fat ratio of 41:42:17; 19.8 kJ/g), a high-fat diet (carbohydrate–protein–fat ratio of 41:16:43; 21.4 kJ/g) or a commercial Western diet (carbohydrate–protein–fat ratio of 41:19:41; 21.5 kJ/g). They found no difference in body weight between the germ-free mice or conventional mice on the low-fat diet, however, when exposed to the high-fat diet germ-free mice gained more body weight and fat when compared with conventional mice, and lower energy expenditure. Germ-free mice gained markedly less body fat on the Western diet in comparison to germ-free mice on the high-fat diet. The investigators then examined the fecal composition of the conventional mouse and found it differed between the diets, with *Firmicutes* increased on both the high-fat diet and Western diet with proportional decrease in *Bacteroides*, leading to the conclusion that the absence of microbiota does not provide protection from diet-induced obesity and the diet composition has a significant impact on the microbial composition.

### Prebiotics

Prebiotics are non-digestible polysaccharides that stimulate the growth of bacteria within the digestive system, namely *Bifidobacterium* and *Lactobacillus*. By doing so, prebiotics promote SCFA production and promote gut barrier function (8). Cani et al. evaluated the effect of *Bifidobacterium* in modulating the inflammatory tone and the development of insulin resistance and obesity (29). They found that the addition of *Bifidobacterium* antagonized the pro-inflammatory state produced by the gut microbiota in response to a high-fat diet, which would normally predispose an individual to insulin resistance and obesity. Everard and colleagues sought to investigate the effect of prebiotic administration in obese and diabetic mice (30). They found prebiotic administration decreased the *Firmicutes* and proportionally increased the *Bacteroides* populations in genetically susceptible mice. In addition, they found that prebiotics improved glucose tolerance, reduced adiposity, and low-grade inflammation, which led the authors to conclude that modulation of microbiota with prebiotics, improves energy homeostasis in obese and diabetic mice. A recent meta-analysis by da Silva and colleagues dissected 61 original articles describing the relationship between the microbiota and obesity and the possible impacts of prebiotics and probiotics (31). The authors found the main effect of associated weight loss was related to an increase in *Bifidobacteria*.

Oligofructose is a prebiotic agent fermented by a number of colonic bacteria used to stimulate the grown of beneficial bacteria (32). Parnell and Reimer evaluated the effects of oligofructose supplementation on body weight and concentrations of ghrelin and PYY as a measure of satiety in overweight and obese adults (33). They randomized 48 healthy adults (BMI >25 kg/m^2^) to receive either oligofructose (21 g/daily) or placebo (maltodextrin) for 12 weeks. They found a reduction of 1.03 ± 0.43 kg in patients with oligofructose as compared to an increase of 0.45 ± 0.31 kg (*p* = 0.01). In addition, they found a lower area under the curve for ghrelin (*p* = 0.004) and a higher area under the curve for PYY with oligofrutcose (*p* = 0.03), which coincided with a reduction in caloric intake and insulin concentrations (*p* ≤ 0.05). This led authors to conclude that oligofructose supplementation has a potential benefit in promoting weight loss as well as improving glucose regulation in overweight and obese adults.

### Probiotics

Probiotics are live microorganisms administered in attempt to reconstitute the gut microbiota, namely *Bifidobacterium*, *Lactobacillus*, *Saccharomyces*, *Streptococcus*, and *Enterococcus*. To date, most clinical trials reported use a combination blend of various microorganisms, which make it difficult to extrapolate, which may be beneficial, and which may be potentially harmful. Several case reports have implicated probiotics as a culprit of severe adverse effects in critically ill patients, including fungemia (*Saccharomyces boulardii*) (34–36), and thus should be used with caution in critically ill and immunocompromised patients.

Ma and colleagues sought to evaluate the effect of probiotics on obesity, steatosis formation, and insulin resistance. In this study, wild-type mice were exposed to either normal or high-fat diets with some receiving VSL#3 (a probiotic mixture of three strains of *Bifidobacterium* and four strains of *Lactobacillus*) (37). They found a high-fat diet to deplete nature killer T-cells with subsequent steasosis formation and insulin resistance. Those mice exposed to probiotic therapy improved the high-fat diet-induced natural killer T-cell depletion and as a result improved steatosis and insulin resistance.

Kakooda et al. evaluated the effects of *Lactobacillus* (*LG2055*) on abdominal adiposity in adults with obese tendencies (38). The investigators randomized 87 subjects (BMI 24.2–30.7 kg/m^2^; abdominal visceral fat area 81.2–178.5 cm^2^) to received either fermented milk with (*n* = 43) or without (*n* = 44) LG2055 for 12 weeks. They found that those receiving LG2055 had a significant reduction (*p* < 0.01) in abdominal visceral and subcutaneous fat areas compared to baseline by an average of 4.6%. In addition, body weight, BMI, waist, and hip measurements were significantly decreased (*p* < 0.001) by 1.4, 1.5, 1.8, and 1.5%, respectively. The authors concluded that probiotic LG2055 has a beneficial influence on metabolic disorders by lowering effects on abdominal adiposity, body weight, and other measures.

### Fecal transplant

Fecal microbiota transplantation (FMT) has been utilized for over 50 years, but has recently gained momentum given its high efficacy in eradicating *Clostridium difficile* infection (39). Recently, there have been several trials evaluating the prospect of altering the gut microbiome as a potential for therapy in obesity and the metabolic syndrome. Vrieze et al. evaluated the effects of FMT on insulin sensitivity in individuals with metabolic syndrome (40). Subjects with metabolic syndrome were randomly assigned to groups set to receive small intestinal infusions from lean donors or autologous microbiota. Subjects who received infusions from lean donors were noted to have an increase in insulin sensitivity (glucose disappearance changed from 26.2 to 45.3 mmol/kg/min; *p* < 0.05) and an increase in butyrate-producing intestinal microbiota 6 weeks post transfusion. This led authors to conclude that intestinal microbiota might be developed as a therapeutic agent to increase insulin sensitivity in patients with metabolic syndrome and insulin resistance.

## Obesity: A Low-Grade Inflammatory State

Metabolic alterations resulting in obesity is associated with a low-grade inflammatory state affecting energy homeostasis and glucose metabolism. Recently, Cani et al. described metabolic endotoxemia as a result of microbiota-derived LPS as a trigger involved in the onset and progression of inflammatory and metabolic sequelae (41). They noted adipose tissue F4/80-positive cells and markers of inflammation were markedly increased in mice continuously infused with LPS. These mice also demonstrated insulin resistance, and adipose tissue weight gain similar to mice that were fed a high-fat diet, which led the authors to conclude that metabolic endotoxemia as a result dysregulates inflammatory tone resulting in the onset and progression of metabolic sequelae such as weight gain and diabetes. Van Greevenbroek et al. further delineated the relationship between obesity and the resultant low-grade inflammatory state (42). They suggested that adipocyte necrosis may be the foundation for the pro-inflammatory response in obesity as caloric intake and energy expenditure result in adipocyte hypertrophy, which may be associated with local hypoxia and apoptosis. Hypertrophic adipocytes begin to secrete TNF-alpha in low quantities stimulating a chemotactic response and attracting macrophages in response to the increase in adipocyte turnover.

## Conclusion

**Figure 1:**
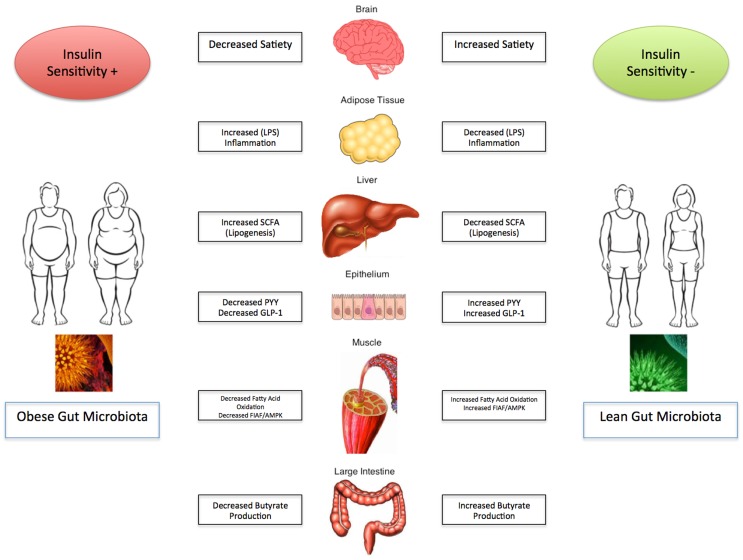
**Gut microbiota and its influence on obesity and the metabolic syndrome**.

Obesity and its related complications are a major detriment on the current state of health care and have significant health care economic implications globally. The evidence presented strongly suggests that the gut microbiota plays a pivotal role in regulating energy homeostasis and the development and progression of obesity and its associated metabolic disorders. It appears that manipulation of the gut flora may be an avenue for potentials of targeted therapy, however, further studies are necessary before implanting them into standard clinical practice. The depth and breadth of the intestinal microbiome remains unknown, and as a result the data presented remains to be established in the clinical realm. Optimal disease state management therefore remains to be defined and remains a significant focus for further clinical investigation. As the “ideal” composition of the gut microbiota starts to unravel, modification must be pursued with caution. The impact of the gut microbiota on obesity and metabolic syndrome is summarized in Figure 1.

## Conflict of Interest Statement

The authors declare that the research was conducted in the absence of any commercial or financial relationships that could be construed as a potential conflict of interest.
